# Insulin Resistance and Coronary Artery Disease: Untangling the Web of Endocrine-Cardiac Connections

**DOI:** 10.7759/cureus.51066

**Published:** 2023-12-25

**Authors:** Fakhar Un Nisa Ashraf, Kashf Ghouri, FNU Someshwar, Sunny Kumar, Narendar Kumar, Komal Kumari, Saira Bano, Saad Ahmad, Muhammad Hasnain Khawar, Lata Ramchandani, Tamara Salame, Giustino Varrassi, Mahima Khatri, Satish Kumar, Tamam Mohamad

**Affiliations:** 1 Medicine, The Royal Wolverhampton National Health Service (NHS) Trust, Wolverhampton, GBR; 2 Endocrinology, Mayo Hospital, Lahore, PAK; 3 Medicine, Liaquat National Hospital and Medical College, Karachi, PAK; 4 Internal Medicine, Burjeel Hospital, Abu Dhabi, ARE; 5 Medicine, New Medical Centre (NMC) Royal Family Medical Centre, Abu Dhabi, ARE; 6 Medicine, Faisalabad Medical College and University, Faisalabad, PAK; 7 Medicine, Shalamar Medical and Dental College, Lahore, PAK; 8 Medicine, Mayo Hospital, Lahore, PAK; 9 Medicine, People's University of Medical and Health Sciences for Women, Nawabshah, PAK; 10 Medicine, Wayne State University, Detroit, USA; 11 Pain Medicine, Paolo Procacci Foundation, Rome, ITA; 12 Internal Medicine/Cardiology, Dow University of Health Sciences, Civil Hospital Karachi, Karachi, PAK; 13 Medicine, Shaheed Mohtarma Benazir Bhutto Medical College, Karachi, PAK; 14 Cardiology, Wayne State University, Detroit, USA

**Keywords:** cardiovascular pathophysiology, metabolic syndrome, atherosclerosis, endocrine-cardiac connections, coronary artery disease, insulin resistance

## Abstract

The relationship between insulin resistance and coronary artery disease (CAD) is a crucial study area in understanding the complex connection between metabolic dysregulation and cardiovascular morbidity. This scholarly investigation examines the intricate relationship between insulin resistance, a key characteristic of metabolic syndrome, and CAD development. The goal is to understand the detailed molecular and physiological connections that underlie the dangerous connection between the endocrine and cardiac systems. The recognition of insulin resistance as a key player in cardiovascular disease highlights the need to study the complex relationships between insulin signaling pathways and the development of atherosclerosis. This research analyzes the molecular processes by which insulin resistance leads to disruptions in lipid metabolism, inflammatory reactions, and malfunction of the blood vessel's inner lining. These processes create an environment that promotes the development and advancement of CAD. As we begin this scientific exploration, it becomes clear that insulin resistance acts as a metabolic indicator and a potent mediator of endothelial dysfunction, oxidative stress, and systemic inflammation. The complex interaction between insulin-sensitive tissues and the vascular endothelium plays a crucial role in defining the pathophysiological landscape of CAD. Furthermore, this discussion highlights the mutual interaction between the endocrine and cardiac systems, where CAD produced by myocardial ischemia worsens insulin resistance through complex molecular pathways. Discovering new therapeutic targets that disrupt the harmful cycle between insulin resistance and the development of CAD shows potential for creating specific therapies to reduce cardiovascular risk in people with insulin resistance. This study aims to clarify the complexities of the connection between the endocrine system and the heart, establishing the basis for a thorough comprehension of how insulin resistance contributes to the development and advancement of CAD.

## Introduction and background

Insulin resistance, a physiological condition marked by reduced cellular sensitivity to insulin, has become a key factor in metabolic illnesses. Metabolic syndrome is a condition that represents a combination of many disorders, such as disrupted glucose regulation, abnormal lipid levels, high blood pressure, and excessive abdominal fat. It is considered a fundamental aspect of metabolic syndrome [1]. The prevalence of insulin resistance is rapidly increasing worldwide, particularly in correlation with rising rates of obesity and sedentary lifestyles [2]. This metabolic abnormality transcends its initial consequences on glycemic management, intricately weaving into the complicated fabric of cardiovascular health, particularly in the context of coronary artery disease (CAD). Insulin resistance, primarily caused by defective insulin signaling pathways, disturbs the intricate equilibrium of glucose management. The condition occurs when cells display decreased responsiveness to the effects of insulin, resulting in an increase in insulin levels as a compensatory mechanism. However, this compensatory reaction indicates metabolic inefficiency, which contributes to the development of metabolic syndrome and its related cardiovascular consequences [2]. Global estimates underline the extensive reach of insulin resistance, offering a troubling portrayal of its prevalence. As societies deal with the challenges of modern lifestyles, the occurrence of insulin resistance has significantly increased, particularly due to the rising rates of obesity and sedentary behavior. This trend is concerning and has reached alarming levels [3].

A thorough comprehension of the implications of insulin resistance, particularly about cardiovascular health, is crucial due to the complex connections it has with many metabolic disorders. CAD is a serious cardiovascular condition characterized by the slow buildup of atherosclerotic plaques in the coronary arteries. This harmful process endangers the blood flow to the heart muscle, resulting in a range of ischemic consequences that can vary from angina to myocardial infarction and heart failure [3]. The widespread presence of CAD worldwide, together with its significant influence on illness and death rates, highlights the need for a more thorough understanding of its complex causes and development. The pathogenesis of CAD is multifaceted, including a complex interplay of genetic, environmental, and metabolic variables. Insulin resistance has been identified as a crucial element in the relationship between metabolic dysregulation and cardiovascular morbidity, leading to thought-provoking concerns concerning the complex linkages between these two conditions [4]. Recognizing CAD not just as a result of mechanical restriction but also as a consequence of systemic metabolic disturbances broadens our conceptualization of its etiology. Despite the progress in understanding insulin resistance and CAD, there is still a noticeable lack of knowledge on the complex relationships between these two conditions. Although increasing evidence suggests a connection between insulin resistance and CAD, the specific processes and intricate interactions between the two conditions are still poorly understood. This review aims to analyze the complex linkages thoroughly, close the knowledge gap, and provide a full synthesis of current views. The complex relationship between insulin resistance and CAD presents a promising area for scientific investigation. However, it still lacks clarity. Although a significant amount of evidence indicates their mutual reliance, a comprehensive comprehension of the fundamental molecular pathways and their causal involvement in coronary atherogenesis is yet unknown [5]. The complex interaction between insulin resistance-induced disruption of lipid metabolism, endothelium dysfunction, and the onset and advancement of atherosclerosis requires thorough investigation and examination [6]. Multiple epidemiological investigations have established connections between insulin resistance and cardiovascular pathology. Nevertheless, establishing a causal relationship based on associations necessitates comprehending the complex molecular and cellular mechanisms that regulate these connections.

This review aims to analyze the intricate relationship between insulin resistance and CAD development by decoding the molecular mechanisms involved. The importance of investigating the complex interrelationships between insulin resistance and CAD goes beyond just academic interest. Understanding the endocrine-cardiac interaction carries crucial implications for clinical practice and therapeutic approaches. Understanding these relationships reveals the underlying causes of CAD. It establishes the basis for innovative diagnostic methods and specific treatments to reduce cardiovascular risk in persons struggling with insulin resistance [6]. The shifting landscape of cardiovascular medicine necessitates a paradigm shift towards a more holistic strategy that emphasizes the delicate connection between systemic metabolic health and cardiovascular outcomes. Expanding our understanding of the causes of cardiovascular disease, we acknowledge the heart as more than just a mechanical pump but rather a complex organ closely connected to the endocrine system [6]. The shift in thinking has significant consequences for tactics aimed at preventing and treating diseases, requiring a comprehensive investigation of the connection between the endocrine system and the heart. This introduction has thoroughly examined the background, frequency, and importance of insulin resistance and CAD. It has established the basis for the following parts by drawing attention to the current lack of knowledge and emphasizing the importance of investigating the connection between the endocrine and cardiac systems. The narrative establishes the foundation for an extensive analysis to elucidate the intricate relationships between insulin resistance and CAD, contributing to therapeutic understanding in this difficult field.

## Review

Methodology

The main objective of this narrative review is to thoroughly investigate the complex relationships between insulin resistance and CAD, clarifying the molecular, physiological, and clinical aspects of their interaction. The scope involves an extensive literature review to combine various viewpoints, identify areas that still need to be addressed, and suggest future research approaches and therapeutic applications. A comprehensive search technique was employed across prominent electronic databases, such as PubMed/MEDLINE, Scopus, and Web of Science. The search encompassed the entire range of databases, from their creation to the most current publications. Medical Subject Headings (MeSH) phrases and related keywords were used to retrieve essential papers. Eligible studies examined the relationship between insulin resistance and CAD, covering molecular processes, epidemiological relationships, clinical symptoms, and therapeutic approaches. Only primary research publications, systematic reviews, meta-analyses, and seminal texts were considered. Studies that did not closely align with the review objectives lacked relevance to the endocrine-cardiac interface or were not published in English were eliminated. Although extensive efforts were made to examine thoroughly, inherent limits exist. Some such constraints include the presence of publication bias, variations in study techniques, and the dynamic character of the area. Furthermore, omitting publications in languages other than English may result in a bias towards English. Since this study pertains to synthesizing pre-existing research, it does not entail the direct inclusion of human or animal subjects. Ethical considerations involve properly utilizing data, guaranteeing accurate attribution, and compliance with ethical rules governing academic research and publication.

Insulin resistance: a multifaceted perspective

Insulin resistance, a primary disturbance in how cells respond to insulin, goes beyond its impact on maintaining glucose balance and affects different aspects of metabolic function. This section examines the molecular complexities of insulin resistance, explicitly investigating the disruption of insulin signaling pathways, the effects on glucose metabolism and cellular function, and the broader metabolic implications related to abnormalities in lipid metabolism and the involvement of inflammation.

Molecular Basis of Insulin Resistance

Insulin, a hormone crucial for regulating metabolism, exerts its effects through complex signaling pathways that coordinate glucose uptake, lipid metabolism, and cellular development. The dysregulation of several pathways characterizes the molecular foundation of insulin resistance. Upon attaching to its receptor on target cells, insulin initiates an intricate network of signaling cascades. The insulin receptor activates downstream components, including insulin receptor substrate (IRS) proteins, phosphoinositide 3-kinase (PI3K), and Akt, ultimately promoting glucose absorption and cellular responses [6]. Nevertheless, in cases of insulin resistance, these pathways experience dysregulation, marked by poor phosphorylation of IRS proteins and diminished activation of downstream effectors [6]. This deviation weakens the cellular reaction to insulin, leading to increased glucose levels in the blood. Multiple causes contribute to the disruption of insulin signaling pathways. Within cells, inflammatory substances that promote inflammation, such as pro-inflammatory cytokines (tumor necrosis factor-alpha (TNF-α)), stimulate enzymes called serine/threonine kinases. This stimulation results in the addition of phosphate groups to serine residues on IRS proteins, disrupting the normal functioning of insulin signaling. In addition, consuming excessive nutrients and incredibly saturated fatty acids triggers intracellular stress reactions, further hindering the transmission of insulin signals [7].

Impacts on Glucose Metabolism and Cellular Function

Insulin resistance significantly impacts how the body processes glucose and how cells operate, disrupting the critical equilibrium for cellular stability. Impaired glucose absorption by target tissues, especially the skeletal muscle and adipose tissue, is the fundamental issue in insulin resistance. This results in increased glucose levels in the bloodstream, as the effectiveness of insulin in promoting the movement of glucose into cells is hindered. As a result, pancreatic beta cells react by increasing the production of insulin in order to counteract resistance, resulting in hyperinsulinemia. Furthermore, under normal circumstances, insulin effectively inhibits the generation of glucose in the liver. However, this inhibitory effect is reduced in a state of insulin resistance. Consequently, the liver maintains the release of glucose into the bloodstream, which leads to prolonged high blood sugar levels [7]. Disruption in the regulation of glucose metabolism becomes a defining characteristic of insulin resistance, which paves the way for the onset of type 2 diabetes. In addition to glucose metabolism, insulin resistance leads to cellular damage. Mitochondrial dysfunction, characterized by decreased oxidative phosphorylation, arises due to insulin resistance. This malfunction adds to the production of reactive oxygen species (ROS), triggering oxidative stress. Increased levels of ROS worsen insulin resistance by disrupting the pathways via which insulin signals and increasing inflammation [7]. The cellular ramifications of insulin resistance encompass disrupted lipid metabolism and lipotoxicity, establishing a connection between insulin resistance and cardiovascular diseases.

Metabolic Consequences

Insulin resistance triggers a series of metabolic effects that affect several physiological systems, including lipid metabolism disruptions and lipotoxicity development. Insulin is vital in lipid metabolism as it inhibits the breakdown of fats in adipose tissue and encourages the storage of lipids. Insulin resistance disrupts this delicate equilibrium. Adipose tissue develops resistance to the anti-lipolytic effects of insulin, resulting in an elevated release of free fatty acids (FFAs) into the bloodstream. Increased levels of circulating FFAs contribute to the accumulation of fat in non-adipose tissues, such as the liver and skeletal muscle, which worsens insulin resistance [8]. Lipid buildup in non-adipose tissues leads to the onset of lipotoxicity, characterized by the harmful impact of excessive lipids on cellular activity. The abundance of lipids within cells, specifically diacylglycerols (DAGs) and ceramides, impair the pathways that allow insulin to function correctly, worsening insulin resistance. Lipotoxicity is closely associated with cellular malfunction, such as endoplasmic reticulum (ER) stress, mitochondrial failure, and apoptosis. Lipotoxicity impairs insulin signaling and plays a role in developing atherosclerosis, a critical factor in the connection between insulin resistance and CAD [8]. The pro-inflammatory and pro-atherogenic actions of lipids contribute to the development and progression of vascular disease.

Inflammation and Its Role in Insulin Resistance

Inflammation is crucial in the complex network of factors contributing to insulin resistance. Pro-inflammatory cytokines coordinate an intricate interaction among immune cells, adipocytes, and insulin-sensitive organs, leading to a pro-inflammatory environment. Elevated levels of pro-inflammatory cytokines, including TNF-α and interleukin-6 (IL-6), are observed in insulin-resistant conditions. These cytokines stimulate serine/threonine kinases, adding phosphate groups to serine residues on IRS proteins and disrupting insulin signaling [9]. The interaction between inflammatory pathways and insulin signaling establishes a continuous cycle, promoting insulin resistance and maintaining long-lasting inflammation. Once seen as a passive reservoir for storing energy, adipose tissue is now recognized as an active endocrine organ in insulin resistance. Adipocytes secrete adipokines, biologically active chemicals with endocrine properties that regulate insulin sensitivity and inflammation. The dysregulation of adipokine secretion, marked by increased levels of pro-inflammatory adipokines such as leptin and decreased levels of anti-inflammatory adiponectin, creates an inflammatory milieu in adipose tissue [9]. Insulin-resistant adipose tissue undergoes heightened infiltration of macrophages, resulting in the development of crown-like structures surrounding deceased or deteriorating adipocytes. Macrophages present in these sites secrete inflammatory mediators, which sustain a condition of persistent low-level inflammation. The defective adipocytes lead to the generation of FFAs, which worsens inflammation and insulin resistance in nearby tissues [10].

CAD: pathogenesis and progression

Atherosclerosis, a multifaceted and advancing vascular illness, is the underlying cause of CAD, resulting in the development of atherosclerotic plaques in the coronary arteries.

Role of Insulin Resistance in Initiating and Promoting Atherosclerosis

Insulin resistance plays a crucial role in the development and progression of atherosclerosis, which in turn increases the risk of CAD. The complex interaction between insulin resistance and the development of atherosclerosis is complicated and goes beyond conventional risk factors. Insulin resistance has a role in causing overall metabolic dysfunction, creating conditions that promote atherosclerosis development. Hyperinsulinemia, a reaction to insulin resistance, increases the synthesis of molecules that promote the development of atherosclerosis, such as endothelin-1 and plasminogen activator inhibitor-1 (PAI-1). These chemicals facilitate the occurrence of inflammation, oxidative stress, and the multiplication of vascular smooth muscle cells, which are crucial mechanisms in the formation of atherosclerotic lesions [10]. Furthermore, insulin resistance leads to dyslipidemia, characterized by increased triglyceride levels and decreased high-density lipoprotein cholesterol (HDL-C) levels. This dyslipidemic profile increases the entry of harmful low-density lipoprotein cholesterol (LDL-C) into the arterial wall, forming foam cells filled with lipids in the inner layer of the artery [10]. The fatty streaks that form indicate the initial phases of atherosclerosis, signifying the beginning of CAD development. Insulin resistance exacerbates atherosclerosis by compromising the anti-inflammatory effects of insulin. Typically, insulin has anti-inflammatory effects by preventing the production of adhesion molecules on the cells that line blood vessels and decreasing the movement of specific immune cells into the inner layer of arteries [10]. In insulin-resistant conditions, the ability of these anti-inflammatory mechanisms to function appropriately is diminished, creating a pro-inflammatory milieu that promotes the development of atherosclerotic plaques. The metabolism of atherogenic lipoproteins is closely connected to insulin resistance, influencing the structure and destiny of lipoproteins in the blood vessels. Insulin resistance causes changes in the way lipoproteins are processed, leading to the creation of small, dense LDL particles. These particles are more likely to undergo oxidative modification and have a more remarkable ability to enter the inner layer of the arteries. In addition, insulin resistance reduces the function of lipoprotein lipase, an important enzyme that breaks down triglycerides in lipoproteins found in the bloodstream. Consequently, there is an increase in the levels of lipoproteins that contain high amounts of triglycerides, which in turn worsens the lipid profile and promotes the development of atherosclerosis. The combined impact of these alterations in lipoprotein metabolism increases vulnerability to lipid peroxidation and the creation of oxidized LDL, a powerful chemical that promotes inflammation and atherosclerosis [11]. Oxidized LDL initiates a series of processes, such as endothelial dysfunction and the attraction of inflammatory cells, which propel the advancement of atherosclerosis.

Endothelial Dysfunction and Vascular Changes

Endothelial dysfunction is a crucial factor in the development of CAD, and insulin resistance has significant effects on endothelial function, ultimately impacting the regulation of blood vessel constriction and contributing to the vascular alterations associated with CAD. The endothelium, a dynamic single layer of cells that covers the inner surface of blood arteries, is vital in regulating vascular homeostasis. Insulin resistance disturbs the intricate equilibrium of endothelial function through multiple routes. An essential result of insulin resistance is reducing insulin's ability to widen blood arteries in resistance vessels. Insulin typically stimulates vasodilation by facilitating the release of nitric oxide (NO) from endothelial cells.

Nevertheless, in conditions of insulin resistance, this reaction is weakened, leading to a decrease in vasodilatory ability [11]. The reduction in the availability of NO is caused by multiple factors, including a reduction of the generation of NO induced by insulin and an increase in the breakdown of NO by ROS formed in conditions of insulin resistance. Insulin resistance additionally amplifies the synthesis of endothelin-1, a potent vasoconstrictor, while diminishing the availability of prostacyclin, a vasodilator. This disparity in vasoactive substances further tilts the balance towards the narrowing of blood vessels, promoting a vascular environment conducive to atherosclerosis development. In addition, insulin resistance triggers persistent low-level inflammation in the endothelium. Adipokines, inflammatory cytokines, and oxidative stress combine to form a favorable milieu for endothelial dysfunction. Endothelial cells increase the expression of adhesion molecules, which helps attract immune cells into the artery intima [12]. The invasion of immune cells paves the way for the development of atherosclerotic plaques. Vasomotor dysfunction, defined by abnormal control of vascular tone, is a crucial feature of CAD pathogenesis. Insulin resistance is closely linked with vasomotor dysfunction, which plays a role in the development of atherosclerosis and the symptoms of CAD. In conditions of insulin resistance, the dysfunction of vasomotor function is demonstrated by the impairment of vasodilation that is dependent on the endothelium. The impaired capacity of the endothelium to produce NO in reaction to different stimuli, such as acetylcholine, highlights the seriousness of vasomotor dysfunction. This compromised NO-mediated vasodilation also affects the coronary arteries, leading to decreased blood flow in the coronary arteries and increasing the likelihood of ischemia events in patients. Insulin resistance hampers the ability of the endothelium to dilate blood vessels and also worsens the body's response to constricting blood vessels. The heightened synthesis of endothelin-1, in conjunction with heightened receptiveness to vasoconstrictor stimuli, further tilts the equilibrium towards vasoconstriction [12]. The disruption of vascular tone leads to an environment that promotes the development of atherosclerosis and facilitates its progression. Additionally, insulin resistance has a role in coronary microvascular dysfunction, a condition defined by the reduced ability of the coronary arteries to expand and decreased dilation of blood vessels that are not dependent on the endothelium. The presence of microvascular dysfunction, which is frequently seen even when there is no blockage in the main coronary arteries, highlights the direct influence of insulin resistance on the heart's blood vessels [13].

Endocrine-cardiac interactions

The complicated interaction between the endocrine and circulatory systems is a complex network of interconnections that goes beyond conventional frameworks. This section examines the reciprocal interaction between insulin-sensitive tissues and the heart, focusing on two-way communication, the influence of insulin resistance on the structure and function of the heart, and the involvement of oxidative stress and inflammatory mediators in shaping the interactions between the endocrine system and the heart.

Bidirectional Communication Between Insulin-Sensitive Tissues and the Heart

Insulin, well-known for its essential function in maintaining glucose balance, significantly impacts the cardiovascular system by interacting with insulin-sensitive tissues. Insulin promotes glucose absorption and controls lipid metabolism in insulin-sensitive tissues like the skeletal muscle and adipose tissue. The metabolic signals triggered by insulin in these tissues play a role in maintaining overall metabolic balance and, importantly, affect the functioning of the heart. The heart, a highly active organ in terms of metabolism, depends on the continuous provision of energy substrates. Glucose is the primary energy source, particularly during heightened demands like exercise or stress [13]. Insulin enhances the absorption of glucose in heart muscle cells, hence impacting the generation of energy and the ability to contract. In contrast, the heart communicates with insulin-sensitive organs by releasing cardiokines, bioactive chemicals generated by cardiac cells. Cardiokines, such as atrial natriuretic peptide (ANP) and brain natriuretic peptide (BNP), affect how insulin works and how glucose is processed in organs far away from the heart. This bidirectional connection is essential for the interaction between the endocrine and cardiac systems, coordinating metabolic responses throughout the body to adapt to the changing needs of the circulatory system. Insulin resistance disturbs the intricate interaction between insulin and the heart, causing significant impacts on the anatomy and function of the heart. The heart, which relies heavily on insulin-mediated glucose uptake, encounters metabolic difficulties in insulin resistance. Insulin resistance reduces glucose uptake and utilization in cardiomyocytes due to poor insulin signaling [13]. The change in metabolism causes the heart to increasingly depend on fatty acid oxidation to provide energy, resulting in a modified preference for substrates. Insulin resistance, characterized by consistently high levels of FFAs in the bloodstream, worsens the buildup of lipids in the heart, a condition called cardiac lipotoxicity. Cardiac lipotoxicity plays a role in forming cardiac steatosis and initiates a series of events, such as impaired mitochondrial activity, increased oxidative stress, and inflammation in cardiomyocytes. The subsequent damage to the heart muscle cells and programmed cell death impair the structure and function of the heart, leading to the development of heart failure. This is a severe outcome of long-term insulin resistance [14]. Furthermore, insulin resistance causes changes in the structure of the heart, characterized by an increase in size and the development of fibrous tissue. The initiation of intracellular signaling pathways, such as the mammalian target of rapamycin (mTOR) pathway, is crucial in facilitating these alterations in structure. Insulin resistance leads to maladaptive changes in the form of the heart, which negatively affects its capacity to relax and fill with blood. This condition, known as diastolic dysfunction, is a critical factor in the development of heart failure with preserved ejection fraction (HFpEF).

Oxidative Stress and Inflammatory Mediators

Insulin resistance affects both the structure and function of the heart. Also, it creates conditions that promote oxidative stress and inflammation in the heart, which in turn affects the interactions between the endocrine and cardiac systems. Oxidative stress, which refers to an imbalance between the creation of ROS and the body's ability to defend against them with antioxidants, plays a crucial role in causing cardiac dysfunction in individuals with insulin resistance. Insulin resistance causes a malfunction in the mitochondria of heart muscle cells, resulting in an elevated formation of ROS due to abnormal activity in the electron transport chain [14]. The ROS, such as superoxide anion and hydrogen peroxide, cause oxidative alterations to cellular macromolecules, triggering a series of events that lead to cardiomyocyte injury. In addition, the activation of nicotinamide adenine dinucleotide phosphate (NADPH) oxidase, a significant enzymatic producer of ROS, is increased in situations of insulin resistance. The continuous release of ROS from NADPH oxidase intensifies oxidative stress, resulting in a harmful feedback loop. Oxidative alterations to lipids, proteins, and DNA in the cardiac cells lead to cellular malfunction and programmed cell death, creating an environment that promotes heart disease. Insulin resistance is characterized by persistent, low-level inflammation, which substantially impacts heart health. Cytokines and chemokines, which are inflammatory molecules, coordinate an intricate interaction between insulin-sensitive organs and the heart. Elevated levels of pro-inflammatory cytokines, such as TNF-α and IL-6, are observed in insulin-resistant conditions and have essential functions in mediating inflammation in the heart [15]. These cytokines have a direct effect on cardiomyocytes, leading to a decrease in their ability to contract and relax properly. In addition, they stimulate the activation of inflammatory signaling pathways, such as the nuclear factor-kappa B (NF-κB) pathway, in cardiac cells. Activating the NF-κB pathway triggers the transcription of genes that promote inflammation, leading to chronic inflammation in the heart. The persistent inflammatory response leads to adverse changes in the structure and function of the heart, including cardiac remodeling, fibrosis, and reduced contractility. In addition, inflammatory cells invade the heart tissue, intensifying the inflammatory environment and worsening cardiac dysfunction. Insulin resistance-induced oxidative stress and inflammation disturb the endocrine-cardiac interface, which plays a role in the complex development of cardiac diseases such as heart failure and myocardial infarction.

Clinical manifestations and outcomes

Insulin resistance, previously limited to metabolic dysfunction, has become a notable cardiovascular health risk factor. This section examines the epidemiological evidence that links insulin resistance and CAD. It also discusses the creation of risk stratification models and the therapeutic implications for managing patients and predicting their prognosis.

Epidemiological Evidence Linking Insulin Resistance to CAD

The extensive epidemiological evidence highlights the connection between insulin resistance and CAD, establishing insulin resistance as a pivotal factor in the development of cardiovascular illnesses. Multiple extensive investigations have repeatedly shown a robust correlation between insulin resistance and a heightened risk of CAD. The Framingham Heart Study, a significant and long-term investigation, discovered that insulin resistance, as evaluated by the homeostatic model assessment for insulin resistance (HOMA-IR), was autonomously linked to an increased likelihood of cardiovascular events. This association remained significant even after accounting for conventional risk factors like hypertension and dyslipidemia [15]. The Multi-Ethnic Study of Atherosclerosis (MESA) found evidence of a direct correlation between insulin resistance and subclinical atherosclerosis, highlighting the gradual connection between insulin resistance and CAD. Moreover, insulin resistance is involved in the development of type 2 diabetes, a widely recognized risk factor for CAD. Prospective studies have repeatedly shown that insulin resistance occurs before the onset of diabetes and is linked to a higher risk of CAD in both diabetic and non-diabetic persons. In addition to quantitative measurements of insulin resistance, qualitative factors such as hyperinsulinemia and poor insulin signaling also increase cardiovascular risk. The Insulin Resistance Atherosclerosis Study (IRAS) emphasized that hyperinsulinemia is an independent predictor of incident CAD, indicating the need to consider insulin resistance and compensatory hyperinsulinemic responses. Incorporating insulin resistance into risk stratification models has become crucial for practical cardiovascular risk assessment. Multiple scoring systems and predictive models have been created to improve risk categorization and optimize clinical decision-making. The Framingham Risk Score (FRS) is a commonly used model that calculates the likelihood of experiencing cardiovascular events within 10 years. Integrating insulin resistance indicators, such as HOMA-IR, into the FRS has enhanced risk prediction, especially in patients who do not have conventional risk factors [16]. Moreover, when high-sensitivity C-reactive protein (hs-CRP), a marker of inflammation, is incorporated into the Reynolds Risk Score, it has been observed that the predicted accuracy is improved, particularly when combined with measurements of insulin resistance. Recent findings also support incorporating imaging techniques, such as coronary artery calcium scoring, to enhance risk categorization in persons with insulin resistance. The MESA found that detecting coronary artery calcium using computed tomography greatly enhanced the ability to predict the risk of heart disease in people with insulin resistance, going beyond the usual risk variables [16]. Advanced models, such as the Prospective Cardiovascular Münster (PROCAM) algorithm and the Reynolds Risk Score Plus, highlight the changing nature of risk assessment. These models integrate a more comprehensive range of risk factors, such as indicators of insulin resistance, to offer a more thorough examination of cardiovascular risk.

Clinical Presentation and Complications

Insulin resistance plays a significant role in the unique clinical presentation of CAD, affecting the symptoms of the disease and impacting its consequences. Understanding these subtle distinctions is crucial for successfully handling patients and predicting their future outcomes. Insulin resistance is associated with the formation of atherosclerosis, which is the main characteristic of CAD, and it also affects the distribution of coronary artery involvement. People with insulin resistance generally show widespread and fast development of coronary atherogenesis, defined by the distribution of atherosclerotic plaques throughout the body and a tendency for these plaques to be more prone to damage [16]. Furthermore, insulin resistance has a role in the emergence of coronary microvascular dysfunction, a disease characterized by compromised vasodilation and disrupted coronary flow reserve. The presence of microvascular dysfunction, which can occur even in the absence of obstructive epicardial CAD, is a contributing factor to angina pectoris. This dysfunction is a distinct manifestation of cardiac pathology associated with insulin resistance. The clinical manifestations of CAD in persons with insulin resistance are varied and may encompass unusual symptoms, such as shortness of breath and exhaustion, in addition to the normal anginal symptoms. A worldwide case-control study has shown that there is a connection between insulin resistance and a higher likelihood of experiencing a heart attack, highlighting the significance of insulin resistance in the manifestation of acute coronary syndromes [17]. The interaction between insulin resistance and conventional risk factors adds more complexity to the clinical scenario. Insulin resistance worsens dyslipidemia, which is marked by high levels of triglycerides and low levels of HDL-C. This contributes to a lipid profile that promotes the development of atherosclerosis. Furthermore, insulin resistance frequently occurs alongside hypertension, resulting in a combined impact that speeds up the advancement of atherosclerosis and increases the likelihood of negative cardiovascular events. Acknowledging insulin resistance as a cardiovascular risk factor requires customized patient care and prediction strategies. Effective management techniques should not solely focus on conventional risk factors but should also aim to address the fundamental insulin-resistant condition. Lifestyle adjustments, such as engaging in regular physical activity and implementing nutritional interventions, are crucial in effectively controlling insulin resistance and reducing the risk of cardiovascular complications. Physical activity increases the body's ability to respond to insulin, improves fats in the blood, and helps maintain a healthy weight. These factors are all important in reducing the risk of cardiovascular disease [18]. Studies have demonstrated that dietary therapies, such as the Mediterranean diet, can improve insulin resistance and lower the occurrence of cardiovascular events. Pharmacological treatments, such as metformin and thiazolidinediones (TZDs), which improve the body's response to insulin, have been studied to determine if they can provide cardiovascular advantages for those with insulin resistance. Metformin, which is commonly used as an initial treatment for type 2 diabetes, has been shown to have cardiovascular benefits that go beyond controlling blood sugar levels. These benefits include enhancing the function of the blood vessel's inner lining and decreasing cardiovascular events [18]. Despite concerns about their safety profile, TZDs have shown potential benefits in select groups with insulin resistance. It is crucial to have a comprehensive approach that considers the overall effects of insulin resistance when managing it. Given the reciprocal communication between insulin-sensitive tissues and the heart, therapies should go beyond regulating blood sugar levels and address the whole metabolic environment instead. Predicting outcomes in persons with insulin resistance requires incorporating cardiovascular risk scores, imaging techniques, and biomarkers. Coronary artery calcium scoring shows potential as a predictive tool in this specific group. The Heinz Nixdorf Recall study showed that coronary artery calcium scores can provide additional predictive value in people with diabetes, indicating its potential usefulness in improving risk assessment in adults with insulin resistance [18]. In addition, newly identified biomarkers, such as indicators of oxidative stress and inflammation, have the potential to provide valuable information about the underlying causes of disease and aid in predicting outcomes. hs-CRP, a biomarker indicating widespread inflammation, has shown predictive significance in forecasting cardiovascular events in persons with insulin resistance. To summarize, acknowledging insulin resistance as a cardiovascular risk factor has significant consequences for the care and prognosis of patients. Optimizing outcomes in patients with insulin resistance and CAD requires tailored methods that specifically target lifestyle factors, use pharmaceutical therapies carefully, and integrate improved risk assessment technologies.

Therapeutic strategies and interventions

To effectively address insulin resistance and its complex association with CAD, a comprehensive strategy is necessary, which includes making changes to one's lifestyle and implementing pharmaceutical treatments. This part focuses on the present therapeutic approaches, assessing the influence of lifestyle modifications on the body's responsiveness to insulin and examining pharmaceutical substances that address insulin resistance and cardiovascular results.

Lifestyle Interventions and Their Impact on Insulin Sensitivity

The primary focus in addressing insulin resistance is lifestyle therapies, which prioritize dietary adjustments, physical activity, and weight management. Adhering to a well-rounded and nourishing diet is crucial for enhancing insulin sensitivity. The Mediterranean diet, which includes a variety of fruits, vegetables, whole grains, and healthy fats, has been proven to improve insulin sensitivity and lower the risk of cardiovascular disease. Limiting the intake of refined carbohydrates and added sugars is essential because these elements in our diet lead to increased blood sugar levels after meals and insulin resistance [18]. Moreover, dietary patterns that facilitate weight loss, such as low-calorie diets or those that prioritize portion management, enhance insulin sensitivity. Including dietary fiber from sources such as legumes and whole grains aids in reducing postprandial glucose fluctuations, which promotes glycemic control [18]. Consistent exercise is a fundamental aspect of managing insulin resistance, as it positively impacts glucose metabolism and cardiovascular well-being. Both aerobic exercise and resistance training improve insulin sensitivity. To achieve optimal metabolic health, it is recommended to participate in a minimum of 150 minutes of aerobic activity each week at a moderate level and engage in strength training activities at least twice a week. Engaging in physical activity stimulates the movement of glucose transporters to the outer layer of the cell, making it easier for skeletal muscle cells to take in glucose. In addition, physical activity improves the functioning of mitochondria, hence decreasing the levels of oxidative stress and inflammation linked to insulin resistance [18]. Weight loss, especially in persons who are obese, has a significant impact on insulin sensitivity. Even a slight decrease in weight has been demonstrated to enhance the body's response to insulin and lower the likelihood of experiencing cardiovascular events. Bariatric surgery, in cases of severe obesity, not only causes significant weight loss but also results in notable enhancements in insulin sensitivity and metabolic parameters. The interaction between lifestyle factors highlights the significance of a comprehensive strategy in addressing insulin resistance. Combining dietary adjustments, physical activity, and weight control produces a synergistic impact, effectively managing several aspects of the insulin-resistant condition [19].

Pharmacological Interventions Targeting Both Insulin Resistance and Cardiovascular Outcomes

Pharmacological therapies are essential for controlling insulin resistance. Various drugs have been proven effective in enhancing insulin sensitivity and reducing cardiovascular risk. Metformin, an initial choice for treating type 2 diabetes, is a fundamental component in the pharmacological strategy for combating insulin resistance. Metformin principally decreases glucose synthesis in the liver, increases glucose absorption in peripheral tissues, and raises insulin sensitivity in the skeletal muscle [19]. In addition to managing glucose levels, metformin has other actions that can assist the cardiovascular system. The United Kingdom Prospective Diabetes Study (UKPDS) showed that metformin treatment in persons with type 2 diabetes resulted in a decrease in cardiovascular events and overall mortality. It is advisable to contemplate metformin treatment for patients with insulin resistance, even if they do not have diabetes, because of its beneficial cardiovascular characteristics, as suggested by the American Diabetes Association. TZDs, such as pioglitazone and rosiglitazone, enhance the body's response to insulin by stimulating the peroxisome proliferator-activated receptor-gamma (PPAR-γ). These drugs promote glucose absorption in adipose tissue and skeletal muscle while exhibiting anti-inflammatory properties [20]. Pioglitazone has specifically shown cardiovascular advantages by decreasing the likelihood of significant adverse cardiovascular incidents in persons with insulin resistance and pre-existing cardiovascular disease. Nevertheless, the need for cautious evaluation arises when administering TZDs due to concerns surrounding possible detrimental consequences, such as fluid retention and bone fractures. Sodium-glucose cotransporter 2 (SGLT2) inhibitors, a more recent category of antidiabetic drugs, have demonstrated potential in targeting insulin resistance and improving cardiovascular outcomes. These medicines mainly block glucose reabsorption in the kidneys, producing glucose excretion in the urine and losing calories. Empagliflozin and canagliflozin, which are inhibitors of the SGLT2, have shown positive effects on cardiovascular health, such as a decrease in the risk of cardiovascular death and heart failure incidents. The Cardiovascular and Renal Outcomes with Empagliflozin in Heart Failure trial and the Dapagliflozin and Prevention of Adverse Outcomes in Heart Failure trial emphasize the potential efficacy of SGLT2 inhibitors in treating cardiovascular outcomes in individuals with insulin resistance and heart failure [20,21]. Glucagon-like peptide-1 (GLP-1) receptor agonists, such as liraglutide and semaglutide, improve glycemic management and insulin sensitivity by increasing insulin secretion, reducing glucagon release, and slowing down stomach emptying. Furthermore, GLP-1 receptor agonists have exhibited advantageous effects on the cardiovascular system. The Liraglutide Effect and Action in Diabetes: Evaluation of Cardiovascular Outcome Results (LEADER) study demonstrated a decrease in significantly adverse cardiovascular events when using liraglutide. In the eValuation of ERTugliflozin effIcacy and Safety CardioVascular outcomes trial (VERTIS-CV) study, semaglutide showed cardiovascular safety but did not meet the non-inferiority criterion for substantial adverse cardiovascular events in participants with vascular disease and type 2 diabetes. Pharmacological therapies that address both insulin resistance and cardiovascular outcomes provide a comprehensive strategy for addressing individuals who are at risk for CAD. Nevertheless, the choice of pharmaceuticals should consider the unique characteristics of each patient, any existing medical conditions they may have, and the possible adverse effects of the medications [21].

Emerging Therapeutic Avenues

As our comprehension of insulin resistance and CAD advances, researchers are exploring new therapy approaches and experimental medications to tackle the complex interrelationships between these conditions. Researchers are now investigating several drugs targeting specific pathways associated with insulin resistance to discover new insulin sensitizers. Researchers are currently studying dual PPAR agonists, which activate both PPAR-α and PPAR-γ at the same time, to determine if they can enhance insulin sensitivity and improve cardiovascular outcomes [21]. Moreover, investigating new targets, such as adiponectin and fibroblast growth factor-21 (FGF-21), shows potential for creating treatments that improve insulin sensitivity and reduce cardiovascular risk. Adiponectin, an adipokine with insulin-sensitizing and anti-inflammatory characteristics, represents a promising target for pharmaceutical therapies to enhance metabolic well-being. Malfunctioning mitochondria significantly influence insulin resistance, and scientists are investigating mitochondrial modulators as prospective targets for treatment. Coenzyme Q10 (CoQ10) and nicotinamide riboside, which boost mitochondrial function, are currently being studied to determine their potential to enhance insulin sensitivity and metabolic parameters [21]. The advent of personalized medicine signifies a transition towards customizing therapeutic approaches according to particular patient attributes, genetic elements, and distinct pathophysiological mechanisms. Personalized medicine approaches have the potential to provide more precise and effective therapies in the setting of insulin resistance and CAD. Genomic research progress has revealed genetic variations linked to insulin resistance and cardiovascular risk. Hereditary profiling enables the identification of individuals with an inborn inclination towards insulin resistance, allowing for tailored therapies and closer monitoring [22]. Additionally, the use of pharmacogenomic factors helps guide the choice of pharmaceuticals according to an individual's genetic profile, enhancing the effectiveness of treatment and reducing adverse side effects. Genetic variations that impact drug metabolism, particularly those that alter cytochrome P450 enzymes, affect the efficacy and safety of pharmacological treatments. Precision medicine utilizes sophisticated imaging techniques, such as coronary artery calcium scoring and cardiac magnetic resonance imaging, to customize risk prediction models based on specific patient features. The accuracy of risk stratification is improved by integrating imaging data with genetic, metabolic, and clinical characteristics, which in turn helps drive individualized therapy options [22]. Customizing therapies according to the specific attributes of insulin resistance, such as its influence on the structure and function of the heart, allows for a more refined and personalized approach to patient management.

Future directions in research and knowledge gaps

Research Frontiers

Within the dynamic realm of biomedical research, comprehending the intricate interaction between insulin resistance and CAD is an area of investigation and advancement. Current research trends are marked by state-of-the-art technologies, innovative approaches, and a comprehensive approach to understanding the complexities of this multilayered interaction. The progress in omics technologies, which include genomes, transcriptomics, proteomics, and metabolomics, has introduced a new period of precision medicine. Researchers are utilizing these high-throughput techniques to decipher the genetic characteristics linked to insulin resistance and CAD. Integrative omics analyses provide a thorough understanding of the molecular pathways, genetic variations, and biomarkers that contribute to the development of both insulin resistance and CAD [22]. Genome-wide association studies (GWAS) persist in discovering genetic regions linked to insulin resistance and susceptibility to CAD. Notable advancements have been made in identifying particular single nucleotide polymorphisms (SNPs) associated with insulin sensitivity and coronary atherosclerosis. Transcriptomic investigations have shown disrupted gene expression patterns in tissues resistant to insulin, providing insight into the molecular mechanisms behind cellular responses to insulin changes. Proteomic and metabolomic studies offer valuable information about the fluctuating patterns in protein expression and metabolic pathways linked to insulin resistance and CAD. Identifying distinct protein markers and metabolite profiles can provide useful diagnostic and prognostic information, guiding the development of focused therapies [23]. Incorporating omics data into systems biology frameworks is a rapidly growing field of study. Systems biology techniques view the interactions among molecular components as dynamic networks rather than separate entities. Network medicine, a branch of systems biology, investigates the interconnections between molecular pathways in health and illness. Current research primarily concerns creating thorough interactome maps that depict the communication between insulin signaling pathways and cardiac regulatory networks. The integrative approach offers a comprehensive comprehension of how disruptions in insulin sensitivity propagate through interrelated cellular cascades, impacting cardiovascular well-being [23]. Identifying crucial nodes within these networks can reveal new targets for therapeutic intervention. The combination of advancements in imaging techniques and artificial intelligence (AI) algorithms is revolutionizing our capacity to observe and analyze the structural and functional alterations linked to insulin resistance and CAD. Non-invasive imaging modalities, such as coronary artery calcium scoring, magnetic resonance imaging, and positron emission tomography, provide comprehensive information about the heart and blood vessels' structure, function, and health. AI algorithms can analyze imaging data to identify subtle patterns and predictive signs that traditional analyses may not see. Machine learning algorithms can use extensive datasets to forecast cardiovascular risk by analyzing imaging parameters and individual patient attributes [24]. The use of AI in clinical decision-making shows potential for individualized risk assessment and therapy strategizing.

Unresolved Questions and Knowledge Gaps

Despite notable advancements in contemporary research, various unanswered concerns and gaps in information impede a thorough comprehension of the complex links between insulin resistance and CAD. Although there is increasing evidence connecting insulin resistance to CAD, the specific molecular processes that drive this association are yet unknown. Understanding the intricate relationship between insulin signaling, lipid metabolism, and inflammatory pathways in the development of atherosclerosis is difficult. Understanding the precise molecular mechanisms by which insulin resistance contributes to the formation and advancement of atherosclerotic plaques is essential for developing specific treatment approaches [24]. Further investigation is needed to understand better insulin's impact on vascular function and the health of endothelial cells. To fully comprehend the effect of insulin resistance on CAD pathogenesis, it is crucial to understand how it disturbs the balance of endothelial function and contributes to an environment that is both pro-inflammatory and pro-thrombotic. The current understanding of insulin resistance and CAD needs to include more information on the distinct distinctions between males and females regarding their expression and progression. Recent findings indicate that the cardiovascular effects of insulin resistance may vary between males and females. Hormonal impacts, hereditary factors, and sex-specific fat distribution patterns cause the variations in these variances. Therefore, it is essential to conduct specialized research to develop preventive and treatment strategies that consider gender-specific aspects [24]. Current research offers static observations on the correlation between insulin resistance and CAD. Long-term studies that follow individuals over extended years are crucial for understanding the temporal dynamics of this relationship. Gaining insight into the course of insulin resistance, its effects on cardiovascular health, and the factors that influence the advancement of the disease is crucial for creating therapies that can address different stages of the disease continuum. Although there are risk prediction models that include signs of insulin resistance, these models still need to be improved for individualized risk assessment. To develop accurate predictive models, it is necessary to incorporate several data sources, such as omics data, imaging parameters, and clinical characteristics. This process needs advanced algorithms and thorough validation in diverse populations. Customizing risk prediction algorithms based on individual features and genetic predispositions is essential for improving their accuracy and practicality in clinical settings [24]. There needs to be more in converting research discoveries into successful intervention approaches. Although lifestyle adjustments and pharmaceutical treatments provide potential, it remains challenging to determine the most effective and tailored interventions for patients with insulin resistance and CAD. Efforts focused on dedicated research are needed to optimize therapeutic regimens, investigate combination medicines, and overcome potential obstacles to adherence [24].

Areas Where Further Research Is Needed for a More Comprehensive Understanding

The gut microbiome's role in metabolic health and its potential influence on insulin resistance and CAD is an emerging area of interest. Understanding how gut microbiota composition and function impact systemic metabolism, inflammation, and cardiovascular health may reveal novel therapeutic avenues. Interventions targeting the gut microbiome, such as probiotics and dietary interventions, warrant exploration for their potential in managing insulin resistance [24]. The impact of environmental factors, including pollutants, toxins, and socioeconomic determinants, on the development and progression of insulin resistance and CAD requires deeper investigation. Unraveling the complex interplay between genetic predisposition and environmental exposures is essential for designing public health strategies that address modifiable risk factors at the population level. Patient-centered research that incorporates patient-reported outcomes is crucial for understanding the lived experiences of individuals with insulin resistance and CAD. Exploring the psychosocial aspects, quality of life, and treatment preferences of affected individuals contributes to a more holistic understanding of the disease burden. Patient-reported outcomes also inform the development of interventions that align with patient needs and preferences [25]. Insulin resistance often coexists with a myriad of comorbidities, including obesity, hypertension, and dyslipidemia. Investigating the intersectionality of these conditions and their collective impact on cardiovascular outcomes is paramount. Research that elucidates how the clustering of metabolic derangements influences disease trajectories and treatment responses will inform holistic and patient-centered care strategies. Understanding the socioeconomic determinants of insulin resistance and CAD is imperative for addressing health disparities. Research that explores how social determinants, access to healthcare, and economic factors contribute to the burden of insulin resistance and CAD in diverse populations will guide public health initiatives and policies aimed at reducing health inequities [25].

## Conclusions

This review has consolidated crucial evidence to uncover the intricate relationship between insulin resistance and CAD, highlighting the significant interplay between metabolic dysregulation and cardiovascular health. Insulin resistance, a critical factor in metabolic syndrome, has effects beyond controlling blood sugar levels and plays a complex role in the development of CAD. A complex viewpoint has arisen, encompassing alterations in cellular pathways and bidirectional communication between insulin-sensitive organs and the heart. A thorough investigation is required to understand the complex relationship between insulin resistance-induced disruption of lipid metabolism, endothelium dysfunction, and atherosclerosis onset and advancement. The connections between insulin resistance and cardiovascular disease are well-established. However, we still need a thorough understanding of the specific biochemical pathways and causal roles in the development of CAD. This study provides a detailed analysis, delivering a profound experience that has the potential to change treatment approaches in managing cardiovascular disease in the presence of insulin resistance. The consequences go beyond clarifying the complex linkages, with direct relevance to clinical practice and future research efforts. The acknowledgment of insulin resistance as a primary element of metabolic syndrome emphasizes its crucial significance in cardiovascular risk. Incorporating indicators of insulin sensitivity into risk assessment models offers clinicians a more comprehensive comprehension of individual risk profiles. Lifestyle adjustments, such as implementing dietary recommendations, engaging in physical exercise, and managing weight, are essential interventions that positively impact insulin sensitivity and cardiovascular health. Pharmacological strategies that address insulin resistance and cardiovascular outcomes offer many risk-reducing alternatives. Conducting specific investigations on patient-centered outcomes and the interconnections between comorbidities is essential. This information integration drives us towards significant progress in preventing, diagnosing, and treating insulin resistance and its considerable impact on cardiovascular well-being. The convergence of endocrinology and cardiology presents an opportunity for further investigation, offering the potential for a future where precision medicine integrates harmoniously with patient-centered treatment.
